# Allergic rhinitis management: a Delphi Consensus promoted by the Italian Society of Pediatric Allergy and Immunology (SIAIP)

**DOI:** 10.1186/s13052-024-01824-5

**Published:** 2024-11-28

**Authors:** Michele Miraglia del Giudice, Gian Luigi Marseglia, Diego G. Peroni, Anna Maria Zicari, Giulio Dinardo, Giorgio Ciprandi

**Affiliations:** 1 Department of Woman, Child and General and Specialized Surgery, University of Campania Luigi Vanvitelli, Naples, Italy; 2 Pediatric Clinic, Fondazione IRCCS Policlinico San Matteo, Pavia, Italy; 3 Department of Clinical, Surgical, Diagnostic and Pediatric Sciences, University of Pavia, Pavia, Italy; 4 Pediatrics Department, University of Pisa, Pisa, Italy; 5 Department of Maternal Infantile and Urological Science, Sapienza University of Rome, Rome, Italy; 6 Allergy Clinic, Casa di Cura Villa Montallegro, Via Monte Zovetto, 27, Genoa, 16145 Italy

**Keywords:** Allergic rhinitis, Management, Clinical practice, SIAIP, Delphi Consensus

## Abstract

Allergic rhinitis (AR) is the most frequent IgE-mediated disease, mainly in children and adolescents. Management of AR in the pediatric age may be heterogeneous, and the available guidelines do not adequately consider this issue. As a result, the Italian Society of Pediatric Allergy and Immunology (SIAIP) promoted a Delphi Consensus to define and evaluate the most relevant aspects of AR management in the pediatric setting in Italy. A qualified board of experts prepared a list of statements that a panel of Italian experts voted on using a web platform. Forty-two pediatricians participated. The results showed that all statements had consensus (> 80% of scores 4 + 5). In particular, there was awareness that AR is a type 2 inflammatory disease requiring adequate treatment. Topical drugs should be preferred, as they are better with cycles. Combined antihistamine/corticosteroid is also considered effective and safe in adolescents. In conclusion, AR deserves adequate attention and care. Current medications are safe and effective; treatment should be addressed to dampen type 2 inflammation and relieve complaints.

## Introduction

A previous Italian survey investigated the features of allergic rhinitis (AR) in children and the prevalence of phenotypes proposed by the Allergic Rhinitis and its Impact on Asthma (ARIA) guidelines [1]. This survey involved 35 pediatric allergy centers throughout Italy and included data from 2,623 patients. The results confirmed the adequacy of ARIA classification and treatment failure in patients with severe AR [2].

Successively, the Italian Society of Pediatric Allergology and Immunology (SIAIP) promoted a further survey to update the knowledge on AR in children and adolescents (manuscript submitted). In particular, this survey has directly involved more than 800 primary care pediatricians, thus reflecting the real-world management of AR in children and adolescents. The findings showed that most Italian primary care pediatricians adopted ARIA guidelines, most children complained of moderate-severe symptoms, asthma was common comorbidity, intranasal corticosteroids and oral antihistamines were first-level choices, and intranasal antihistamine plus corticosteroid was a frequent therapeutic option, mainly in subjects with moderate-severe symptoms.

Therefore, these two surveys underscored the importance of obtaining updated and accurate information concerning the practical management of allergic rhinitis in Italian children.

Presently, there are some international guidelines concerning the AR management [2–6]. Despite, this abundance of documents, there are no pediatric-oriented guidelines nor documents specific for the Italian pediatric setting. As a result, the SIAIP performed a Delphi Consensus on the practical management of children with AR. This iterative initiative involved outstanding experts on this topic who discussed and approved a list of statements to administer a group of Italian pediatricians with proven experience in AR management. Namely, the Delphi method was an indirect, anonymous, and iterative way to obtain a consensus [7].

## Materials and methods

### Delphi method

A group of five experts (the authors of this paper) on AR management constituted a steering committee devoted to produce the present Delphi Consensus. This steering committee drafted and shared a questionnaire (first round) to administer to a group of pediatricians who had to express their agreement grade on the statements (second round).

The components of the steering committee have a proven experience on AR management documented by more than 30 years of clinical practice on allergic diseases and scientific value demonstrated by more than 20 publications on this topic produced in the last five years.

The steering committee formulated the statements considering the current scientific literature on AR management and personal expertise.

The group of involved pediatricians was selected based on clinical practice in third-level teaching hospitals and scientific merit documented by at least five publications on this topic produced in the last five years. In addition, all participants are Fellows of the SIAIP and work in all regions of Italy, so that the experts’ panel reflects geographic diversity across Italy.

The first round consisted of a face-to-face interaction to discuss the initial draft of questions and approve them.

The second round consisted of the creation of a specific online platform to collect the vote of participants about the grade of agreement and to assure the anonymity of each participant.

The Delphi Consensus comprised questions concerning the definition of AR and type 2 inflammation, epidemiology, comorbidity, symptoms characteristics, and medications (use and schedules). The Table 1 reports in detail all questions.

**Table 1:** Statements included in the Delphi Consensus on the pediatric allergic rhinitis management in Italy and relevant answers expressed as percentages of participants voting score 4 or 5, and means *±* standards deviations (SD) of the scores 4 and 5

Statement	% agreement (scores 4 + 5)	Mean score (SD)
1) Allergic rhinitis is a disease caused by a dysregulation of the immune system that is characterised by type 2 inflammation.	97.2	4.9 (0.35)
2) Type 2 inflammation of the nasal mucosa is mainly represented by infiltration by eosinophils.	94.5	4.7 (0.46)
3) Type 2 inflammation is closely correlated with sensitisation/re-exposure (inhalation) to the causative allergen, even in the absence of symptoms (minimal persistent inflammation)	91.7	4.7 (0.47)
4) As part of the World Allergy Epidemic, allergic rhinitis is a condition that affects more than 20% of the paediatric population and its prevalence is steadily increasing.	97.2	4.7 (0.46)
5) Allergic rhinitis can no longer be considered a trivial disease, as it is accompanied by asthenia, irritability, depression of mood, anxiety, poor concentration and sleep disturbances, all annoying symptoms that cause a significant negative impact on quality of life.	97.2	4.9 (0.32)
6) Allergic rhinitis is often associated with other conditions such as atopic dermatitis, allergic conjunctivitis, rhinosinusitis, bronchial asthma, eosinophilic oesophagitis, food allergy and sleep disorders. In addition, in paediatric age it can cause altered development of the craniofacial massif and normal development of the dental arch.	100	4.87 (0.35)
7) Allergic rhinitis is the main risk factor for the onset of bronchial asthma and, if already present, the main risk factor for poor asthma symptom control.	97.2	4.7 (0.46)
8) From these considerations, the concept emerges that allergic rhinitis deserves a specific PDTA (Diagnostic Therapeutic Care Pathway) with particular attention to the search for comorbidities (especially asthma).	100	4.8 (0.42)
9) The typical symptoms of allergic rhinitis are nasal itching, sneezing (blanks), watery rhinorrhoea and nasal obstruction. The first three depend mainly on the abundant release of histamine during the allergic reaction (histamine-dependent symptoms), whereas nasal obstruction is mainly an expression of allergic inflammation.	100	4.7 (0.44)
10) It follows from this statement that histamine-dependent symptoms are more responsive to the use of antihistamines, whereas nasal obstruction is more responsive to the use of topical corticosteroids.	94.4	4.8 (0.4)
11) Topical antihistamines are quicker and allow a lower dosage than the systemic route. They may also be effective on possibly associated ocular symptoms.	80.5	4.5 (0.5)
12) Topical corticosteroids are effective and safe drugs at the recommended dosage. They effectively reduce the degree of type 2 inflammation and consequently relieve nasal obstruction and can also act on comorbidities such as rhinosinusitis or eye symptoms or asthma (if associated). At the recommended doses, they are safe even when used for long periods of time.	100	4.7 (0.48)
13) Topical corticosteroids must be administered appropriately, considering the symptomatology and mode of application.	100	4.7 (0.44)
14) A fixed combination antihistamine/corticosteroid (azelastine/fluticasone) available as a nasal spray has been available for some time. Existing literature highlights its high efficacy, rapid action and safety even in paediatric age.	97.2	4.6 (0.48)
15) The azelastine/fluticasone combination acts with a dual effect on both the histamine response and inflammation with greater speed and efficacy than the non-combined administration of the two drugs on all symptoms of allergic rhinitis.	100	4.7 (0.48)
16) The combination of azelastine/fluticasone should be considered in children/adolescents when maximum results are to be achieved in a short time.	91.7	4.6 (0.49)
17) It is preferable to use the azelastine/fluticasone combination for appropriate periods of time (at least one to two weeks) to ensure prompt resolution of symptoms and adequate control of type 2 inflammation.	88.9	4.6 (= 0.49)
18) However, the combination azelastine/fluticasone can also be used in symptomatic mode in the case of sporadic but nevertheless intense rhinitis episodes	81	4.6 (0.51)
19) These considerations give rise to the notion that the combination of azelastine/fluticasone could result in a saving of inhaled corticosteroids when using topical corticosteroids for asthma therapy	80.6	4.5 (0.51)
20) The combination azelastine/fluticasone can lead to savings in the use of oral antihistamines with lower economic costs and greater adherence to treatment, which is particularly relevant in adolescence.	83.4	4.6 (0.49)
21) In both seasonal and perennial allergic rhinitis, the nasal anti-H1/steroid combination may act more rapidly and therefore be preferred.	86.1	4.5 (0.51)
22) In any case, it is essential to take the time to explain well to children/adolescents and their families what allergic rhinitis is, what causes it, and the use of the most appropriate medication, in order to achieve maximum patient engagement in the correct management of the condition.	100	4.9 (0.28)

After collecting and analyzing the second round’s results, the steering committee discussed and approved them.

The Delphi Consensus process was conducted in June 2024.

### Delphi statements

The Delphi document comprised questions concerning the definition of AR and type 2 inflammation, epidemiology, comorbidity, symptoms characteristics, and medications (use and schedules). The Table 1 reports in detail all questions.

### Delphi assessment

The Delphi Consensus Panel was requested to rate their agreement with each questionnaire statement using a 5-point Likert scale, such as 1 (strongly disagreement), 2 (disagreement), 3 (partially agreement), 4 (agreement), and 5 (strongly agreement). Each expert provided individual and anonymous vote on the statements, considering routine practice and clinical evidence. The number and percentage of participants scoring each item was calculated.

The scientific committee then discussed the results in a virtual meeting. For each questionnaire statement, the consensus was considered to have been achieved based on the agreement (sum of score 4–5) of at least 80% of the Consensus Panel and the successive acceptance of the steering committee.

The statistical analysis was descriptive and a mean score of the sum of 4 + 5 scores was calculated also considering the standard deviation.

## Results

The first round served to define a list of statements to administer to the panel of experts designed by the steering committee. This round included five independent experts who constituted the steering committee.

After thorough debate, the agreement among these steering committee members was entire, i.e., a 100% complete agreement (score 5) was reached for all 22 statements.

**Fig. 1:**
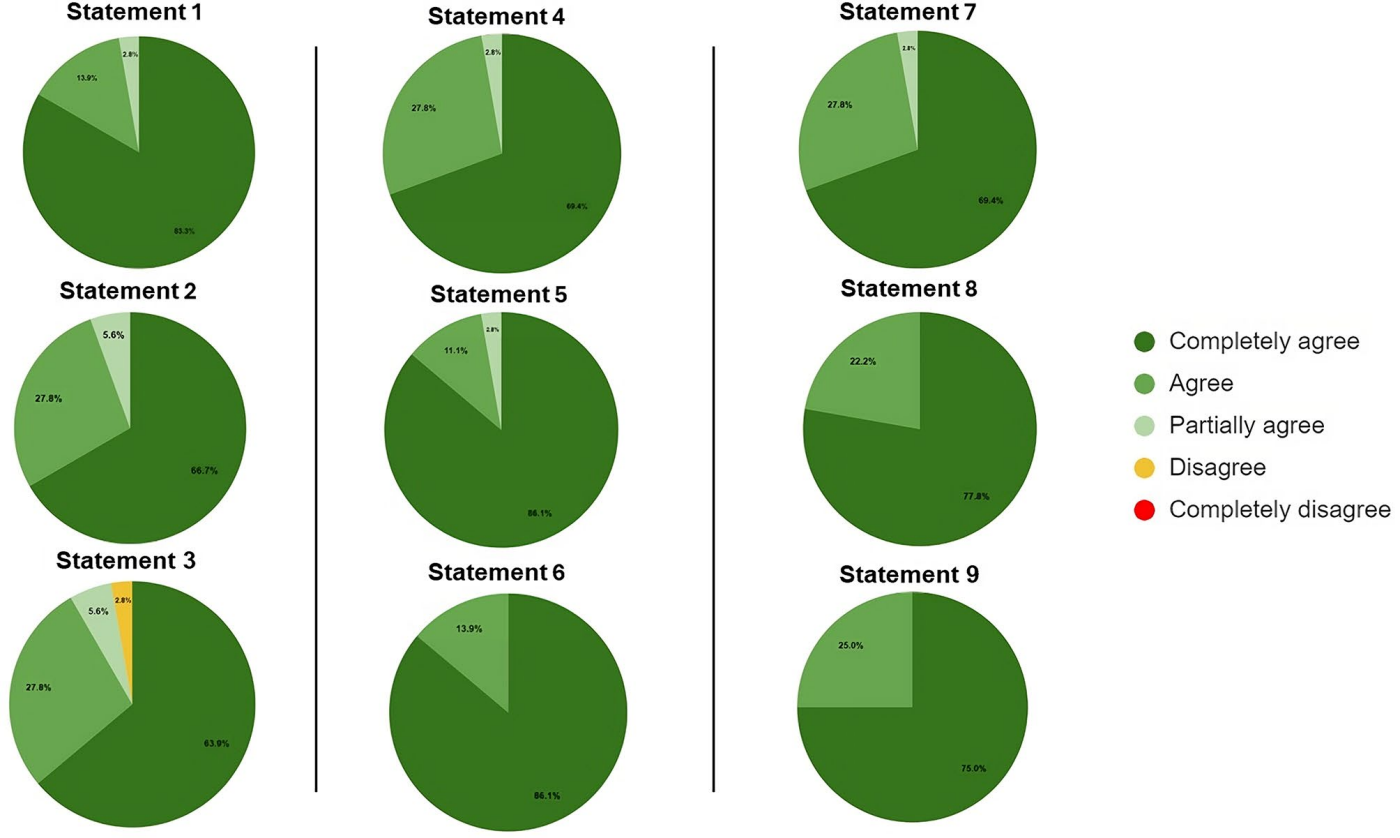
Distribution of agreement score for Statements 1–9

**Fig. 2:**
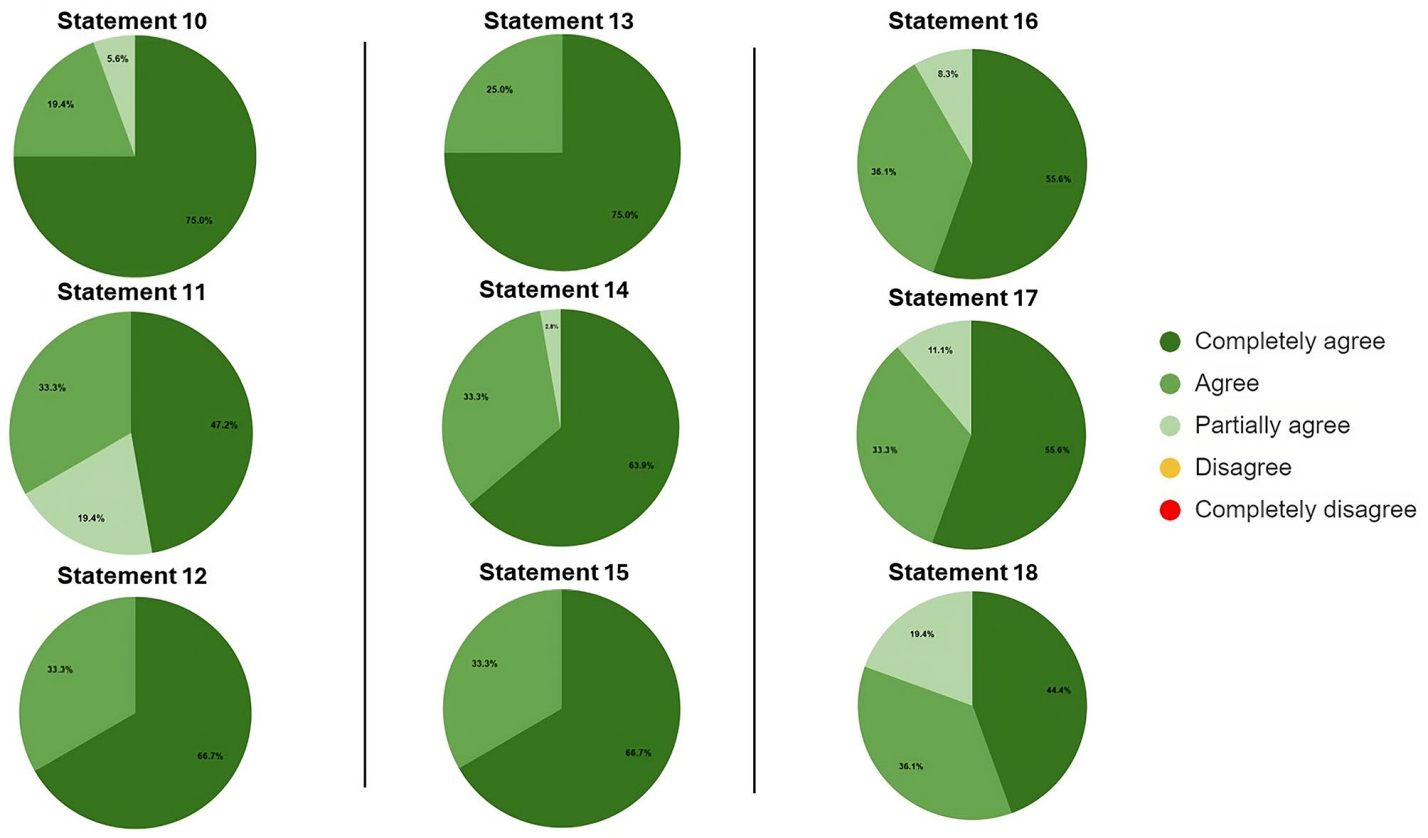
Distribution of agreement score for Statements 10–18

**Fig. 3:**
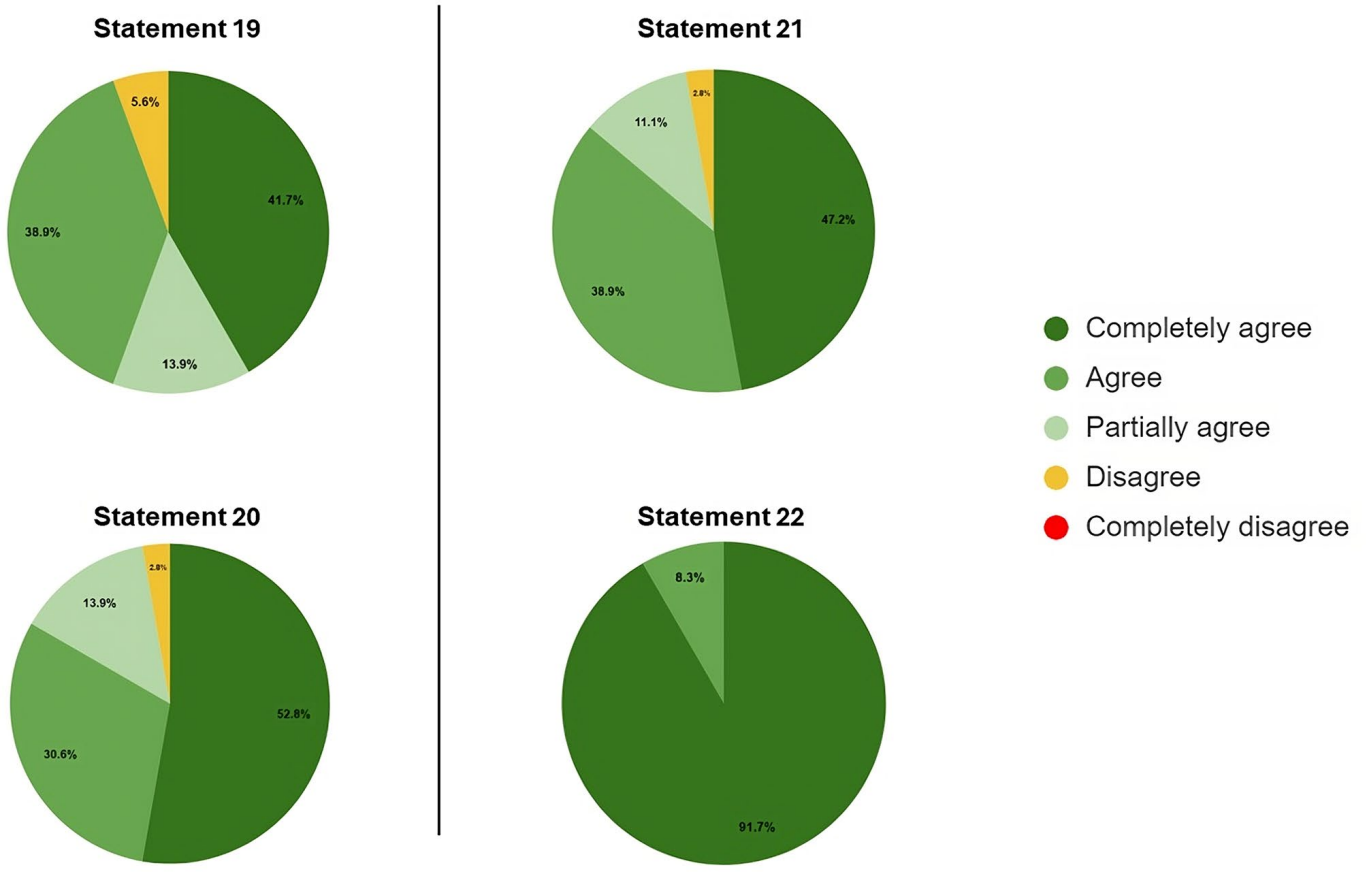
Distribution of agreement score for Statements 19–22

The second round included 42 other experts, identified by the steering committee, who voted on the 22 statements. The voting results are reported in Figs. 1 and 2, and 3.

Seven statements (6, 8, 9, 12, 13, 15, and 22) obtained a full agreement level, such as 100%.

Nine statements (1, 1, 2, 3, 4, 5, 7, 10, 14, and 16) obtained an agreement level between 90 and 99%.

The remaining six statements reached an agreement level between 80 and 89%.

Consequently, all statements reached, as a priori defined, a positive consensus, such as > 80%.

## Discussion

The present Delphi Consensus globally involved 47 Italian experts on AR management in the pediatric setting. Therefore, the present Delphi Consensus reflected how pediatric AR is managed in Italy’s real-world practice. The profile of participants also guaranteed an adequate standard of outstanding scientific profile.

There is good agreement (> 90%) among participants on the concepts that type 2 inflammation signs AR and leads to eosinophilic infiltration of the nasal mucosa. Namely, there is a body of evidence sustaining this concept [8]. In addition, large majority of participants believe that allergic inflammation depends on causal allergen exposure even without symptom occurrence, i.e., the concept of minimal persistent inflammation [9].

Most participants agreed about the increasing prevalence of AR as recently demonstrated by a meta-analysis [10].

Almost all participants (97%) shared the concept that AR should be not considered a trivial disease, as it is accompanied by asthenia, irritability, depression of mood, anxiety, poor concentration and sleep disturbances, all annoying symptoms that cause a significant negative impact on quality of life. The document ARIA and robust evidence confirmed these AR characteristics [11].

There was also full agreement (100%) about the notion that AR frequently presents comorbidity [12]. Namely, AR is often associated with other conditions such as atopic dermatitis, allergic conjunctivitis, rhinosinusitis, bronchial asthma, eosinophilic esophagitis, food allergy and sleep disorders [13]. In addition, in pediatric age, AR can cause altered development of the craniofacial massif and normal development of the dental arch [14].

A near full agreement (97.2%) concerned the idea that AR is the main risk factor for the onset of bronchial asthma and, if already present, the main risk factor for poor asthma symptom control. In this regard, there is large evidence supporting this statement and is widely shared [15]. As a result, all participants agreed about the need of thorough diagnostic pathways to early detect asthma comorbidity. There is evidence that adequately treating AR significantly affect asthma course [16].

There was also full consensus about the pathophysiological characteristics of AR symptoms. Nasal itching, sneezing (blanks), and watery rhinorrhoea depend mainly on the abundant release of histamine during the allergic reaction (histamine-dependent symptoms), whereas nasal obstruction is mainly an expression of allergic inflammation [17]. Instead, nasal obstruction is mainly an expression of allergic inflammation and intranasal corticosteroids efficaciously dampen type 2 inflammatory events [18].

However, an agreement (80.5%) was reached concerning the use of topical antihistamines, probably regarding the possible relief of ocular symptoms. Namely, there is a large pier of studies in fact that have shown that intranasal antihistamines allow significant dose reduction and are more effective than the systemic formulation [19]. In addition, there is also evidence about their efficacy in alleviating ocular symptoms as recently documented by a meta-analysis [20].

There was a full agreement (100) concerning the efficacy and safety of topical corticosteroids in treating patients with AR. There was shared awareness that effectively reduce the degree of type 2 inflammation and consequently relieve nasal obstruction and can also act on comorbidities such as rhinosinusitis or eye symptoms or asthma [21]. Consistently, all participants agreed on the fact that topical corticosteroids must be administered appropriately, considering the symptomatology and mode of application [22].

Almost full consensus (97.2%) regarded the statement declaring that a fixed combination antihistamine/corticosteroid (azelastine/fluticasone) has high efficacy, rapid action and safety even in pediatric age [23]. Similarly, there was full agreement on the concept that azelastine/fluticasone combination acts with a dual effect on both the histamine response and inflammation with greater speed and efficacy than the non-combined administration of the two drugs on all symptoms of allergic rhinitis, as well documented in literature [24]. There was also high grade of consensus (91.7%) about the concept that the combination of azelastine/fluticasone should be considered in children/adolescents when maximum results are to be achieved in a short time. Namely, this fixed combination provides a quick symptomatic activity [25].

Most participants (88.9%) agreed that using the azelastine/fluticasone combination is indicated for appropriate periods of time (at least one to two weeks) to ensure prompt resolution of symptoms and adequate control of type 2 inflammation. This statement reflects the need of assuring a dampening of type 2 inflammation that usually requires one-two weeks [26]. There was also consensus (81%) about the combination azelastine/fluticasone can also be used in symptomatic mode in the case of sporadic but nevertheless intense rhinitis episodes. In this case, some participants preferred to prioritize inflammation control activities over merely symptomatic ones.

Moreover, there was an agreement about the notion that the combination of azelastine/fluticasone could result in a saving of inhaled corticosteroids when using topical corticosteroids for asthma therapy. Probably, some participants were doubtful that properly treating allergic rhinitis can also positively influence the anti-inflammatory treatment of asthma. In fact, there is a large body of evidence that instead shows how important it is to treat allergic rhinitis well to ensure adequate asthma control [27]. Consistently, some panelists expressed low agreement about the combination azelastine/fluticasone can lead to savings in the use of oral antihistamines with lower economic costs and greater adherence to treatment, which is particularly relevant in adolescence. Actually, there is documentation that azelastine/fluticasone improves the AR management [28].

Also concerning the rapidity of action and consequently the preference for azelastine/fluticasone there was a wide agreement (86.1%). There is evidence that this combination is quicker than antihistamines alone in relieving complaints [25].

The last statement gathered full approval as to take the time to explain well to children/adolescents and their families what allergic rhinitis is, its causes and the use of the most appropriate medication, in order to achieve maximum involvement (patient engagement) in the proper management of the disease is crucial.

The present document had some limitations, including the collection of personal opinions, the lack of objective measures, and mostly the absence of clinical data. Moreover, the statements concerned only some aspects of AR management. However, this consensus involved outstanding pediatricians managing many children with AR with large experience. Thus, the results provided robust outcomes that also reflected what happens in the real world. Further studies should confirm these findings, adopting adequate methodology. In the future, this initiative could involve a wider audience of pediatricians involved daily in their clinical practice in the management of children and adolescents with AR. Moreover, the SIAIP is currently engaged and will be even more so in the future in initiatives aimed at updating knowledge on the topic through various educational initiatives (distance learning, meetings, courses, and congresses). The primary outcome should be to achieve a large application of these recommendations in clinical practice.

In conclusion, the present Delphi Consensus reported that a panel of Italian expert pediatricians considered the type 2 inflammation the leading characteristic of allergic rhinitis, so deserving adequate treatment. Contextually, this documented endorsed the concept that a rapid symptom relief represents a priority objective in managing children and adolescents with allergic rhinitis. In addition, safety should be always evaluated prescribing any therapy. In this context, the present Delphi Consensus underlined the experts’ opinion that the fixed combination of intranasal corticosteroid plus antihistamine (i.e., azelastine/fluticasone) may represent a valuable option for treating young people with allergic rhinitis. This issue reflects what the most recent guidelines advocate on AR management.

## Data Availability

All data generated and analyzed during this Delphi Consensus are included in this published article.

## Abbreviations

AR: Allergic rhinitis
ARIA: Allergic rhinitis and its impact on asthma
SIAIP: Italian Society of Pediatric Allergology and Immunology
